# Integration of natural selection across the life cycle stabilizes a marine mussel hybrid zone

**DOI:** 10.1002/ece3.11086

**Published:** 2024-03-10

**Authors:** Allison B. Willis, Evgeniya Ermolaeva, Amaelia Zyck, Rhiannon Rognstad, Shannon Davis, Thomas J. Hilbish

**Affiliations:** ^1^ Department of Biological Sciences University of South Carolina Columbia South Carolina USA

**Keywords:** complex life cycle, directional selection, hybrid zone, larvae, marine mussels

## Abstract

Hybridization among related species is now recognized as common but it remains unclear how hybrid zones persist for prolonged periods. Here, we test the hypothesis that selection in different components of the life cycle may stabilize a hybrid zone. A hybrid zone occurs in southwest England between the marine mussels *Mytilus edulis* and *M. galloprovincialis*. Previous studies have found strong directional selection against alleles from *M. edulis* occurs among hybrids in the adult stage. Traditional hybrid zone models argue that alleles that are selected within the hybrid zone are replaced by migration from neighboring parental population into the hybrid zone. In this system, however, migration occurs out of this hybrid zone into neighboring parental populations. This hybrid zone should therefore be unstable and dissipate, yet this zone has persisted for more than 30 years. We tested and rejected the hypothesis that differences in fecundity may select for *M. edulis* alleles within this hybrid zone and thus counter the selection observed against these alleles among adults. We also tested the hypothesis that selection during the larval stage may counter selection against *M. edulis* alleles in the adult stage. We found that selection favors *M. edulis* alleles during the veliger stage of larval development. The direction and strength of selection during the larval stage are sufficient to counter strong selection during the adult portion of the life cycle. This hybrid zone is stabilized by opposing forms of directional selection operating in different portions of the life cycle.

## Graphical abstract

We tested the hypothesis that selection in different components of the life cycle may stabilize a hybrid zone in southwest England between two species of marine mussels. We rejected the hypothesis that differences in fecundity select for *Mytilus edulis* alleles within this hybrid zone and thus counter the selection observed against these alleles among adults. We demonstrate that selection favors *M. edulis* alleles during the larval stage of development which is sufficient to counter strong selection during the adult portion of the life cycle and results in the stabilization of this hybrid zone.

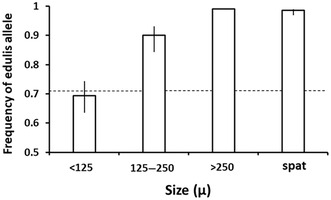

## 1 INTRODUCTION

Hybridization among related species is now recognized as a prevalent process (Schumer et al., 2016). Ongoing hybridization is a common feature of modern biodiversity but there is also increasing evidence of ancient hybridization events that are preserved in the genomes of extant species (Moran et al., 2021). Despite the prevalence of hybridization, there remain many questions about how modern hybrid zones may persist for indefinite periods and how genomes are “stabilized” following hybridization (Schumer et al., 2016). These may be related as the longer a hybrid zone persists the greater the opportunity for stable combinations of genes to arise through recombination and selection. Traditional models of hybridization argue that hybrid zones may be stable if selection against hybrid genotypes occurs within the zone, and this is offset by migration from neighboring parental populations into the zone. Selection against hybrids may be due to either the incompatibility of the two genomes (Barton & Hewitt, 1985) or mismatches between hybrid genotypes and the environmental gradient across which the hybrid zone occurs (Harrison & Rand, 1989). Current reviews, however, suggest that the dynamics of hybrid zones and the forms of selection that operate within them may be considerably more complicated (Moran et al., 2021; Schneemann et al., 2020; Schumer et al., 2016).

An excellent example of the potential complexity of genetic and environmental processes that may stabilize a hybrid zone is the extensively studied hybridization between the mussels *Mytilus edulis* and *M. galloprovincialis* in the eastern Atlantic. In this region, multiple hybrid zones naturally occur between these two sister species (Bierne, Borsa, et al., 2003; Coustau et al., 1991; Fraïsse et al., 2014; Hilbish et al., 1994, 2002; Skibiniski et al., 1983). There are additional hybrid zones between these two species of anthropogenic origin (Simon et al., 2020). The best studied of these hybrid zones occurs in southwest England. This hybrid zone has persisted for at least 30 years (Gardner, 1994; Hilbish et al., 2002; Skibiniski et al., 1983; Wilhelm & Hilbish, 1998), with little evidence of either contraction or expansion. This hybrid zone clearly violates the concepts incorporated into traditional hybrid zone models that seek to explain the persistence of hybrid zones. Strong directional selection occurs among adults within the hybrid zone that favors genotypes with alleles from *M. galloprovincialis* (Gardner et al., 1993; Gardner & Skibinski, 1988; Gilg & Hilbish, 2003a, 2003b; Hilbish et al., 2002; Skibinski & Rodrick, 1991; Wilhelm & Hilbish, 1998). This is contrary to the expectation that selection operates against individuals with hybrid genotypes (Barton & Hewitt, 1985). Typically, the frequency of *M. edulis* alleles among newly settled mussels is ~0.95, and over several years selection reduces the frequency of these alleles to <0.30 (Gilg & Hilbish, 2003a, 2003b; Wilhelm & Hilbish, 1998). Thus, selection is both directional and intense. In addition, rather than dispersal occurring from parental populations into the hybrid zone, as expected in traditional models, this hybrid zone is trapped between two barriers to dispersal such that migration predominantly occurs out of the hybrid zone into bordering parental populations (Gilg & Hilbish, 2003a, 2003b) and there little if any migration from parental populations into the hybrid zone. While not as intensively studied, there is evidence that other hybrid zones that occur within the region are similar to the hybrid zone in southwest England in that they are trapped between barriers to dispersal and selection is predominantly directional in favor of *M. galloprovincialis* alleles (Gardner et al., 1993, Hilbish et al., 2012, pers. obs.).

Given the directional selection against *Mytilus edulis* alleles within the southwest England hybrid zone and the isolation of the zone that prevents replenishing these alleles from neighboring parental populations this hybrid zone should not be stable. Rather, in the time during which this zone has been observed, it should have evolved to feature a low frequency of alleles from *M. edulis*. Other mussel populations in the region have this structure; they typically have a high frequency of *M. galloprovincialis* alleles and a frequency of *M. edulis* alleles of around 5% (Hilbish et al., 2002, 2003; Skibiniski et al., 1983). Skibiniski et al. (1983) suggested that the genetic structure of these populations may be the result of ancient hybridization that has equilibrated at a low frequency of *M. edulis* alleles because of directional selection. Nonetheless, it remains unclear why many of these hybrid zones remain stable over protracted periods of time.

Here, we test the hypothesis that there are other forms of selection that occur in other life‐stages of marine mussels that counter the directional selection against *M. edulis* alleles observed in the adult portion of the life cycle. Marine mussels have complex life cycles that include a sedentary adult phase and a free‐swimming larval stage. Consequently, adult and larval life‐history stages are ecologically independent of one another, including alternative mechanisms for feeding and movement (Bayne, 1976). During development a mussel undergoes radical metamorphosis several times, which almost certainly includes large changes in patterns of gene expression (Durland et al., 2021) that may result in altered patterns of selection.

We test two general hypotheses for possible counter‐selection that restores the high frequency of *Mytilus edulis* alleles among newly settled juveniles. The first is that adults with *M. edulis* alleles may be more fecund than individuals with a high frequency of *M. galloprovincialis* alleles. Mussels devote progressively more energy to reproduction as they grow larger (Gardner & Skibinski, 1990). It is possible that *M. edulis*‐like individuals either consistently devote more effort to reproduction or they have a greater asymptotic fecundity as adults than do *M. galloprovincialis*‐like individuals. We test this hypothesis by comparing the total biomass devoted to reproduction across the entire size range of adult individuals of different genotypes from a hybrid zone population. The second hypothesis we test is the possibility that during the larval stage there is selection in favor of *M. edulis* alleles. To test this hypothesis, we measured allele frequencies among larval mussels in different portions of their time in the plankton. This hypothesis argues that there is selection during the larval phase that favors *M. edulis* alleles and is sufficiently strong to counteract the strong directional selection against these alleles observed in the adult portion of the life cycle. If either of these hypotheses is supported, it will indicate that selection in hybrid zones may be further complicated by the possibility of different forms of selection operating in different portions of the life cycle.

## 2 METHODS AND MATERIALS

### 2.1 Reproductive analysis

**FIGURE 1:**
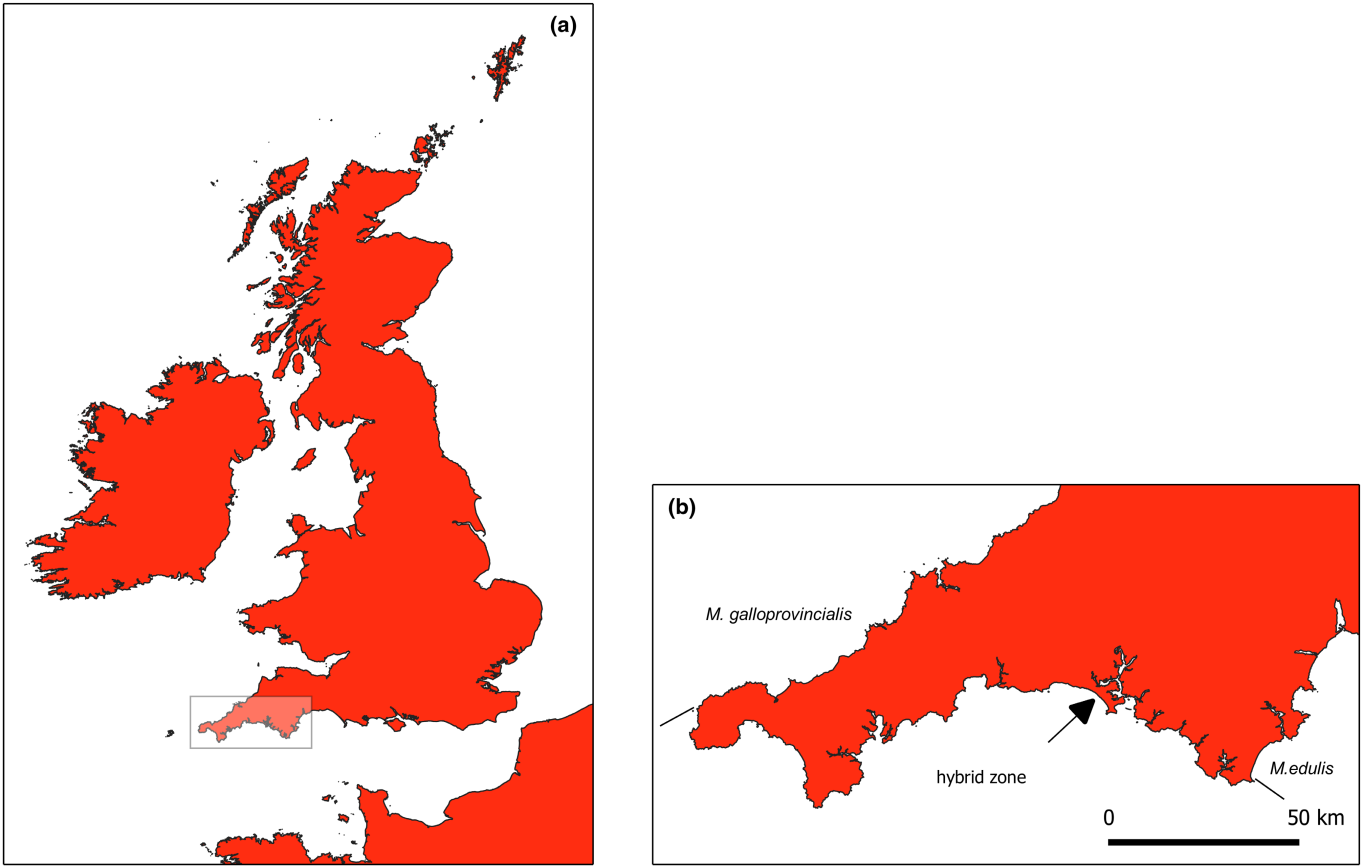
Panel (a) depicts the location of the hybrid zone between *Mytilus edulis* and *M. galloprovincialis* in the United Kingdom used in this study. Panel (b) depicts southwest England and the position of the two species and their hybrids. The arrow indicates the sample location in Whitsand Bay used in this study.

In early May 2017, approximately 160 adult *Mytilus* individuals were collected from mid‐tidal locations on Whitsand Bay on the coast of Cornwall in southwest England (Figure 1). Samples were collected in May because previous studies indicate that during this time individuals would be at their peak reproductive condition but will not have yet released gametes (Secor et al., 2001). Whitsand Bay was chosen as a sample site because previous work has demonstrated it to be very representative of the genetic structure of the hybrid zone in southwest England (Hilbish et al., 2002). Individuals collected ranged in size from 17 to 48 mm. Shell length was measured for each individual and then it was dissected. The full mantle, which includes reproductive tissue, was extracted from each individual and weighed to obtain the wet mantle weight. Then, the remaining tissue was extracted, weighed, and added to the mantle weight to obtain the total tissue weight. From the extracted mantle of each individual, a piece of tissue approximately 0.25 cm^2^ in size was collected for histological analysis. This piece of tissue was preserved in 10% Baker's formalin. For genetic analysis, a small piece of the mantle edge was collected and preserved in 95% ethanol.

The mantle tissue samples for histological analysis were processed according to the protocol of Secor et al. (2001). The samples were then embedded in paraffin blocks and sliced with a microtome into 5 μm thick sections. Representative sections of mantle tissue from each sample with the largest areas were mounted on glass slides using a wet mount technique. The slides were then incubated at ~37°C overnight to remove excess water. The slides were stained with hematoxylin and eosin, cover‐slipped, and visualized using a video microscope system. For each individual, five non‐overlapping images of the mantle tissue were taken, representing five independent frames from each mussel. The histological images were then analyzed using ImageJ (Abramoff et al., 2004). A grid of 20 points was overlaid onto each image, yielding a total of 100 points per individual, and using the Cell Counter feature, each point was scored according to the type of cell on which it rested. Cell types that were scored included mature gametes, immature gametes, adipogranular cells, vesicular connective tissue (VCT), and “other,” which included undistinguishable structures, empty space, and other cell types (Secor et al., 2001). In some cases, the tissue was thin and fragmented after histological preparation and fewer points were counted. The number of combined immature and mature gametic hits was divided by the total number of points counted to obtain a gamete volume fraction (GVF) for each individual. The GVF was multiplied by the mantle tissue weight to determine the total weight of gametic tissue for each individual.

Total gametic tissue weight and total tissue weight measurements were imported into RStudio (Version 1.1.414) and separate analyses of co‐variance (ANCOVA) were used to analyze the relationship between total tissue weight, gamete weight, and sex within each homozygous genotype. Heterozygous genotypes were not analyzed due to small sample size. A third ANCOVA was used to analyze the relationship between total tissue weight, gamete weight, and genotype.

### 2.2 Larval sampling and analysis

Larval samples were collected 8 times between May 2 and August 31, 2012, from two locations within Whitsand Bay at stations that were approximately 1 and 3 km offshore (latitudes and longitudes 50.3386 and −4.2693, 50.3207 and −4.2792, 50.3233 and −4.2384, and 50.3182 and −4.2651). Samples were collected using plankton tows to collect 2 m^3^ of water. The samples were preserved in 95% ethanol and brought back to the laboratory at the University of South Carolina for sorting and genetic analysis. Subsamples of 1/200th of the original sample were analyzed under a dissecting microscope and bivalve larvae in different stages of development were collected. Between 5 and 15 subsamples were analyzed for each sample depending on how many individual bivalve larvae were observed. Fewer than 15 subsamples were analyzed if the total number of larvae exceeded 150. Mussel larvae were sorted into three sizes classes: <125, 125–250 and >250 μm. Individual larvae were scored for developmental stage based on the shape of the shell, the presence of an eye spot and size. Larvae were classified as “D‐stage” if they had the distinctive shape of a capital letter D. “D‐stage” larvae are typically less than 48 h post‐fertilization. If larvae no longer had the “D” shape but did not have an eye spot or a larval foot they were classified as veliger larvae. Mussel larvae may remain in the veliger stage for several weeks depending upon temperature and other environmental factors (Bayne, 1976). If a larva had a pigmented “eye spot” and a larval foot, it was classified as a pediveliger larva. Individual larvae were placed in separate tubes for genetic analysis. Shell length measurements were made along the longest axis of the larval shell.

Newly settled individuals (called spat) were collected from Whitsand Bay, near the sites where the plankton sampling occurred following the methods of Gilg and Hilbish (2003a, 2003b). Spat that were <0.375 μm in shell length were assumed to have settled in the previous 5–7 days (Gilg & Hilbish, 2003a).

DNA were extracted from adults using the protocol of Truett et al. (2000). Larval and spat DNA was extracted according to Zhan et al. (2008) and Gilg and Hilbish (2000), respectively. Individuals were genotyped by PCR of the *Glu‐5ʹ* gene, which differs diagnostically between the two parental species, using the protocol of Rawson et al. (1996) except using primers Me‐15 and Me‐16 developed by Inoue et al. (1995). Many species of bivalve larvae are morphologically similar, so prior to genotyping larvae at the *Glu‐5′* gene we determined which individuals were mussels in the genus *Mytilus* using PCR following the protocol of Larsen et al. (2005). Individual larvae that were confirmed to be *Mytilus* were then analyzed via a second PCR assay, using the *Glu‐5′* marker as described above.

## 3 RESULTS

### 3.1 Adult allele frequencies

**FIGURE 2:**
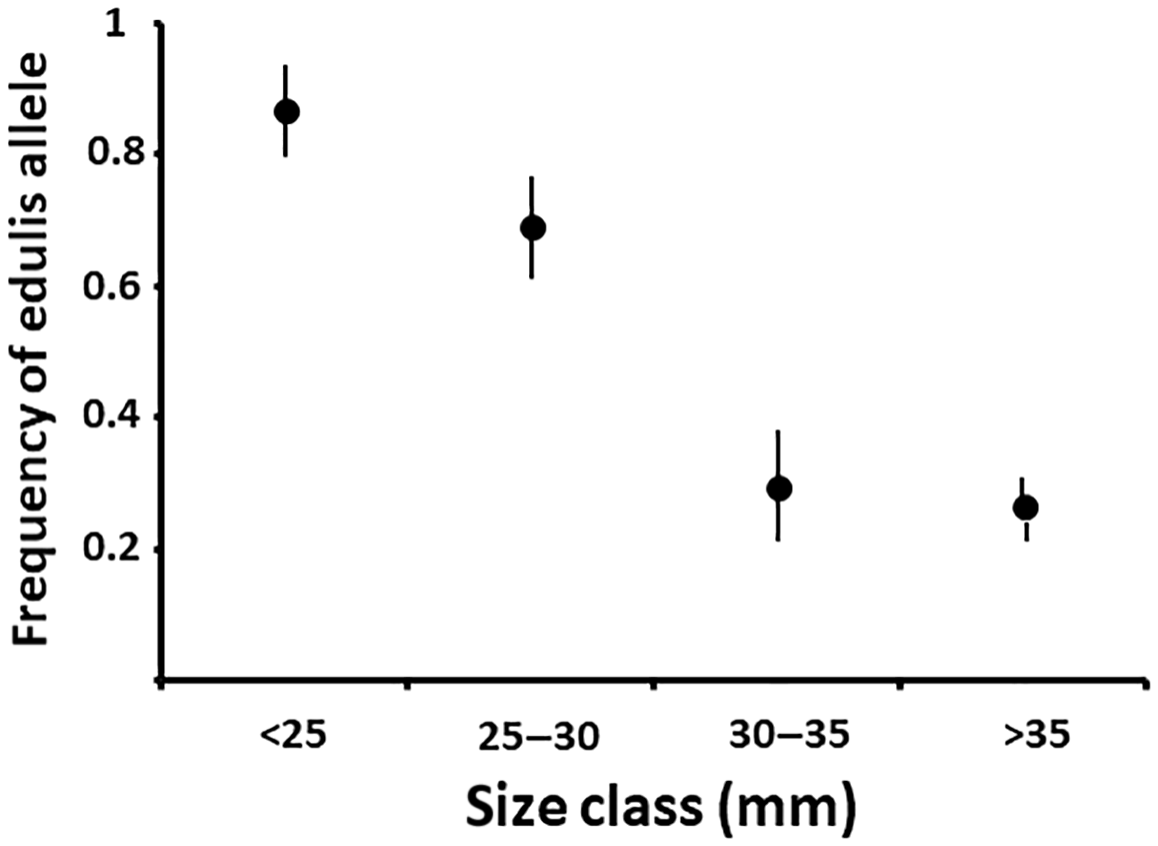
The relationship between allele frequency (frequency of *M. edulis* alleles) and size (in shell length) in adults sampled from Whitsand Bay. Size is given as size categories (<25 includes individuals as small as 17 mm and >35 includes individuals as large as 45 mm). Error bars are 1 SE of the mean.

The frequency of *Mytilus edulis* alleles was strongly dependent upon size (Figure 2). Spat and small adult individuals had *M. edulis* allele frequencies greater than 90% (Figure 4), while individuals over 35 mm in shell length had frequencies of this allele of ~26% (Figure 2). Standard errors of observed allele frequency were calculated using a binomial GLM, which uses logit link function to calculate asymmetric standard errors as the observed values approach 0 or 1. The standard errors are plotted in Figure 2. A *G*‐test of independence showed that the relationship between allele frequency a size was highly significant (*G*_1,3_ = 51.5, *p* < .001). This indicates that the adult population at Whitsand Bay continues to exhibit the same genetic structure indicative of directional selection against the *M. edulis* allele that has previously been documented in this hybrid zone (Hilbish et al., 2002; Skibiniski et al., 1983; Wilhelm & Hilbish, 1998).

### 3.2 Reproductive assessment

**FIGURE 3:**
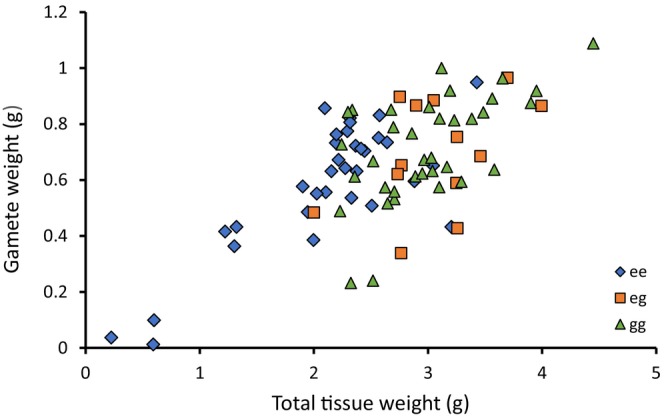
Total weight of gametes as a function of total soft tissue weight of individuals from Whitsand Bay that are homozygous for the *M. edulis* allele (blue diamond), homozygous for the *M. galloprovincialis* allele (green triangle), and heterozygotes (red square).

Gamete weight was analyzed as a function of total tissue weight by sex for each of the homozygous genotypes (*M. edulis* homozygotes and *M. galloprovincialis* homozygotes). The results of the ANCOVA indicate that there was no significant effect of sex on the relationship between gamete weight and total tissue weight for *M. edulis* (*F* = 1.502, df = 1, 29, *p* = .23). The ANCOVA conducted for *M. galloprovincialis* homozygotes showed a slight but significant effect of sex on gamete tissue weight (*F* = 4.282, df = 1,32, *p* = .0467). Males had approximately a 12.5% greater gamete weight than did females, but these differences were minor compared to the nearly four‐fold variation observed among individuals within any given size class (Figure 3). Heterozygotes were not tested for differences in gamete weight by sex because there were too few to provide a meaningful analysis. There was no evidence for a difference in gamete weight between the sexes among heterozygotes.

Because there was found to be either no or only a small difference in reproductive output between the sexes in both the *M. edulis* and *M. galloprovincialis* homozygotes, the data were combined to increase the sample size, and gamete weight was regressed against total tissue weight by genotype to analyze the effect of genotype on fecundity (Figure 3). Heterozygotes were included in this analysis. ANCOVA results showed that reproductive output was strongly dependent upon total body weight (*p* < .0001), but there was no significant effect of genotype on gamete weight once total body weight was considered (*F* = 0.401, df = 2,76, *p* = .671).

### 3.3 Larval genetics

**FIGURE 4:**
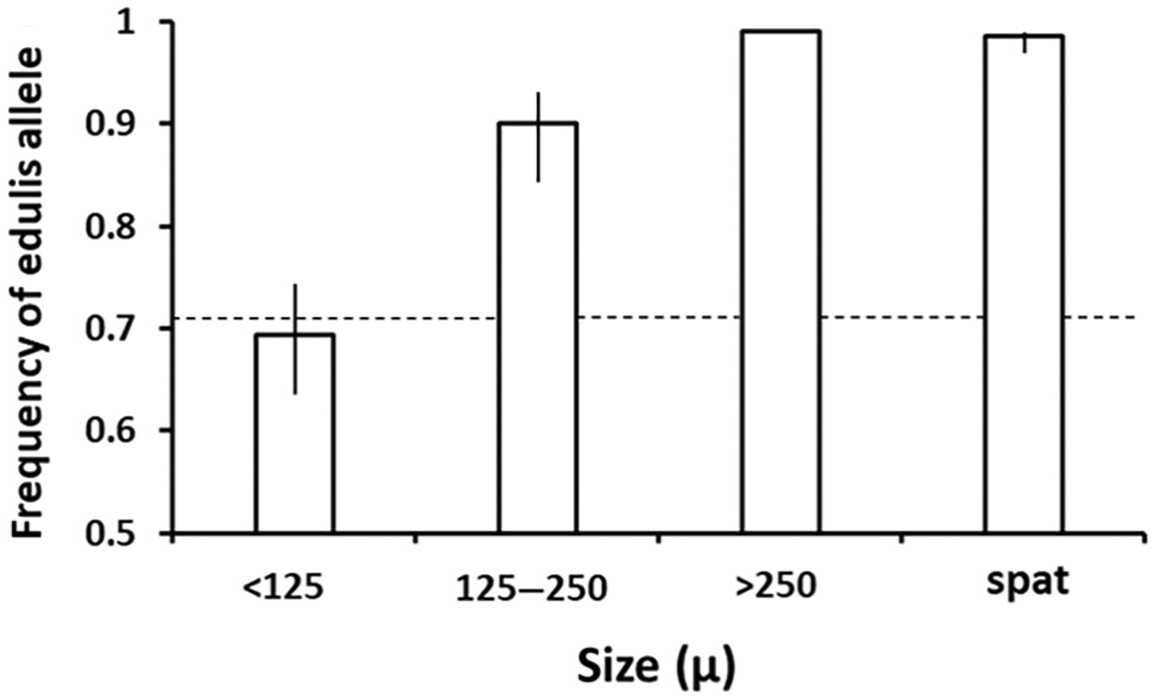
The frequency of *Mytilus edulis* alleles at different stages of larval and post‐larval development. The “spat” category includes newly settled post‐larvae individuals. The dashed line depicts the expected allele frequency among newly fertilized larvae assuming random mating and no selection as calculated by Gilg and Hilbish (2003b). Error bars are 1 SE of the mean. Since the observed frequency of edulis alleles in the >250 size class is 1 the SE is undefined and is not plotted.

Larvae that were <124 μm in length were almost all in the D‐stage, indicating that they had completed metamorphosis from the trochophore to the veliger stage within the last 24–48 h. Larvae that were 125–250 μm in length were all veligers. Larvae that were >250 μm in length were almost all pediveligers, indicating that they had recently metamorphosed from veliger to pediveliger and were prepared to settle and continue metamorphosis to a spat. Mussel larvae with a shell length of <125 μm had a *Mytilus edulis* allele frequency of 0.694 (Figure 4). Individuals in the range of 125–250 μm had an allele frequency of 0.897 and those in the >250 μm range had a *M. edulis* allele frequency of 1. Newly settled spat (<375 μm) had an allele frequency of approximately 0.98 (Figure 4). Standard errors of observed allele frequency were calculated using a binomial GLM which uses logit link function to calculate asymmetric standard errors as the observed values approach 0 or 1. A *G*‐test showed that the variation in allele frequency among different size classes was significance at *p* < .001 (*G* = 46.8 with df = 3). *G*‐values cannot be calculated if the allele frequency in any of the treatment groups is 1, so to calculate *G* we added 1 *M. galloprovincialis* allele to the >250 μm larval group. This is a conservative adjustment since it makes the variation in allele among treatment groups smaller than what was observed. We used an exact test for goodness of fit using the GWASExactW Package in R to compare the observed number of each genotype in each larval and post‐larval size class to the expected distribution of genotypes under Hardy–Weinberg equilibrium. We used an exact test because the sample sizes and/or the expected number for some of the genotypes were <5 and traditional chi‐square or log‐likelihood test are highly biased under these circumstances. We found that in each case there was a significant deficit of heterozygotes observed compared to the expected values (Table 1).

**TABLE 1:** Comparison of observed versus expected number of each genotype among larvae and post‐settlement individuals (=spat).

Size class (μm)	ee (obs/exp)	eg (obs/exp)	gg (obs/exp)	*p*
<125	24/17.34	2/15.29	10/3.37	1.84 × 10^−7^
125–250	25/23.33	2/5.36	2/0.31	.014
“Spat” <375	74/72.99	1/2.98	1/0.03	.020

*Note*: ee = individuals homozygous for the *Mytilus edulis*, gg = individuals homozygous for the *M. galloprovincialis* allele and eg = heterozygotes. obs = observed number of individuals and exp = the number expected under Hardy–Weinberg equilibrium. *p* is the probably that the observed distribution of genotypes departs from the expected distribution using an exact test for goodness of fit. The larval size class of >250 μm has been omitted because the frequency of the e allele was 1 (the sample size for this group was 22 individuals).

## 4 DISCUSSION

In this study we assessed two hypotheses that may explain the stabilization of the mussel hybrid zones observed in southwest England and other locations within Europe. The first of those is that *Mytilus edulis* may have greater fecundity than does *M. galloprovincialis* which may explain the initially high frequency of *M. edulis* alleles among newly settled mussel spat. We found no significant difference in fecundity among genotypes nor was there any evidence that *M. edulis* continued to invest more heavily in gamete production with increasing size compared to *M. galloprovincialis*. Our results agree with those of Secor et al. (2001), who found no evidence for differential reproductive output among hybrid genotypes. Secor et al. (2001) tested whether selection against *M. edulis*‐like genotypes was the result of differential reproductive investment and consequently they restricted their assessment to individuals in intermediate size classes because those are most strongly affected by directional selection against *M. edulis* alleles (Wilhelm & Hilbish, 1998). In this study we included the full range of available sizes so that we could also test the hypothesis that *M. edulis* has a greater asymptotic investment in reproduction with increasing size compared to *M. galloprovincialis*. There was no evidence of any difference in reproductive investment among genotypes.

Gilg and Hilbish (2003b) took into account the size structure of hybrid populations, allele frequency among size classes, and size‐specific fecundity to calculate the expected frequency of *M. edulis* alleles at the time of fertilization. They assumed that fecundity was equivalent among genotypes and our study confirms that assumption. With no difference in fecundity, they estimated the *M. edulis* allele frequency at the time of fertilization would be 0.71. To increase that frequency to 0.9 would require *M. edulis* to be at least three times more fecund than *M. galloprovincialis*. A difference of this magnitude would have been easily detected; thus, we conclude that there is no differential reproductive output among hybrid genotypes and conclude that differential fecundity is not a stabilizing force of the hybrid zone in southwest England.

The second hypothesis that we examined is that counterselection may occur during larval development. As described above, Gilg and Hilbish (2003b) estimated that the expected frequency of *Mytilus edulis* alleles at the time of fertilization is 0.71. In contrast the frequency of this allele among new recruits in the southwest England hybrid zone is typically 0.95 (Gilg & Hilbish, 2003a, 2003b). Thus, our second hypothesis attributes this discrepancy to selection in favor of *M. edulis* occurring during the larval development cycle, the period between fertilization and recruitment.

There could be five periods throughout larval development during which selection may favor *M. edulis* alleles: during the transition from trochophore to D‐stage veliger, during the veliger stage, during the metamorphosis from veliger to pediveliger, from pediveliger to spat, or after settlement. Critical developmental events occur during each of these stages of larval development. During periods of radical metamorphosis, altered patterns of gene expression may give opportunity for selection in favor of *M. edulis* alleles. Selection in favor of *M. edulis* alleles within the larval development cycle would counter the selection in favor of *M. galloprovincialis* alleles among adults, and thus, if this hypothesis proves to be correct counterselection could be the force stabilizing the hybrid zone in southwest England.

We found that individuals who were less than 48 hours old in the D‐stage of larval development exhibited a *Mytilus edulis* allele frequency of 0.69, which corresponds very closely to the predicted gamete pool allele frequency of 0.71 (Gilg & Hilbish, 2003b). Thus, at the D‐stage, no selection has yet occurred. There was a significant change in allele frequency during the veliger stage of the larval period. *M. edulis* allele frequency increased from 0.69 to 0.90 among veliger larvae. Pediveligers had a *M. edulis* alleles frequency of 1.0 (Figure 3). There was no significant change in allele frequency between the pediveligers and newly settled spat. Thus, it appears that there is, in fact, directional selection in favor of *M. edulis* alleles during the veliger larval stage. Consequently, we accept our hypothesis of counterselection during larval development being the stabilizing force for the maintenance of the hybrid zone in southwest England, and we identified that the moment during which this counterselection occurs is early in the larval development cycle, prior to the pediveliger stage.

In this study, we used the genetic marker *Glu‐5′* (Rawson et al., 1996) to assess the genetic composition of hybrid mussel populations. *Glu‐5′* is highly differentiated between *Mytilus edulis* and *M. galloprovincialis* and is highly correlated with other genetic markers used to distinguish these two species (Rawson et al., 1996). The decline in *M. edulis* allele frequency observed in this study is correlated with similar changes in allele frequency observed at more than 50 single nucleotide polymorphisms (Diz & Skibiniski, 2023). Consequently, we consider the results we obtained here using the *Glu‐5′* marker to accurately reflect selection across the genome. This creates the question, however, of why a single genetic marker would reflect selection across the entire genome. Diz and Skibinski (2023) have shown that hybrid populations of mussels are often a mixture of nearly “pure” *M. edulis*, “pure” *M. galloprovincialis* and true recombinant hybrids (e.g., backcrosses and multigenerational hybrids). Thus, it seems likely that much of the selection we are observing is due to changes in the frequency of the “pure” types, which differ in their larval and adult phenotypes.

Laboratory breeding studies have attempted to assess whether selection occurs in the larval stage of mussels involved in this hybrid zone, but these have had mixed results. Beaumont et al. (1993) found that F_1_ hybrid larvae had greater mortality but faster growth than did larvae of either *Mytilus edulis* or *M. galloprovincialis*. However, Beaumont et al. (2004) found no evidence of selection at the larval stage and that hybrids had lower growth rates. Beaumont et al. (2004) cautioned that large family‐specific effects may overwhelm differences among genotypes in laboratory culture experiments, especially when only a few parents are used in constructing the crosses. Bierne et al. (2006) is the only study to experimentally generate F_2_ hybrid crosses and backcrosses. They found that larvae from F_2_ crosses suffered greater mortality than did backcrosses (F_1_ × *M. galloprovincialis*) or crosses among *M. galloprovincialis*. Unfortunately, they were unable to spawn *M. edulis* and consequently pure *M. edulis* crosses for backcrosses to *M. edulis* could not be made. Overall, these studies suggest that there may be selection against hybrids between *M. edulis* and *M. galloprovincialis*, but they are inconclusive as to whether selection should favor hybrid individuals with *M. edulis* alleles.

Only one other study has examined the genetics of mussel larvae in nature. Bierne, Bonhomme, and David (2003) sampled late‐stage larvae near the margin of an estuarine population of *M. edulis* and an open‐coast hybrid population with a high frequency of *M. galloprovincialis* alleles. They found clear evidence of two larval pools occurring at different times, each with allele frequencies very similar to the two nearby adult populations. One of the larval populations had a genetic composition very similar to the nearby hybrid population. There was no evidence of selection in favor of *M. edulis* alleles in the late‐stage larvae they sampled. Bierne, Bonhomme, and David (2003) did not sample early‐stage larvae; thus, it is not possible to infer whether there was a change in allele frequency over time. However, their results contrast with ours because in our samples all late‐stage larvae had a high frequency of *M. edulis* alleles.

The source of selection we observed during the veliger stage of development remains unclear, but it is very unlikely that the traits under selection in different portions of the life cycle are related. Adult mussels are sedentary and use a gill to feed, while larval mussels are mobile using a velum for both locomotion and feeding. During the transition from the larval to adult stage the velum is resorbed, and the gill is formed. Previous studies suggest that selection against *Mytilus edulis* alleles during the adult phase of the life cycle related to differences in attachment strength to the substrate (Gardner & Skibinski, 1991; Schneider et al., 2005), which in turn appears to be related to differences in behavior. Adult mussels use byssal threads to attach to the substrate. They can detach the threads and use their foot to move a limited distance. *M. edulis* is more mobile than *M. galloprovincialis* and are more likely to move to the surface or edge of a mussel bed. This behavior makes *M. edulis* have lower strength of attachment to the substrate and more vulnerable to being dislodged by waves. This difference in behavior is believed to be the ecological mechanism by which adult *M. galloprovincialis* are selectively favored in open coastal environments found in the hybrid zone in southwest England.

This mechanism is irrelevant to mussel veliger larvae as they don't have a foot or form byssal threads for attachment to the substrate. The mechanism of selection that favors *Mytilus edulis* alleles during the larval stage is very likely to be unrelated to the mechanism of selection against these alleles during the adult stage. Given the large differences between larval and adult mussels it is not surprising that the mechanism of selection differs among different stages of the life cycle. Durland et al. (2021) and found different patterns of gene expression across larval development in oysters and found indications that selection varied among larval stages [however, also see Hedgecock (2022) and Durland et al. (2022)]. Hua et al. (2023) also found evidence that selection may occur across larval development in the mussel, *Mytilus galloprovincialis*.

We also observed a large heterozygote deficiency among larvae, especially at the D‐stage of development. There are at least two possible explanations for this observation; there may be positive assortative mating among gametes, or the larval population may result from the mixture of two differentiated adult populations. The latter hypothesis is unlikely. D‐stage larvae are 24–48 hours old and are unlikely to have dispersed very far from their parent population. The results of Gilg and Hilbish (2003a) indicate that larvae are unlikely to have dispersed more than 6 km, which would mean that D‐stage larvae collected in Whitsand Bay are likely to be the progeny of the adult populations within Whitsand Bay. Adult populations in other portions of the hybrid zone have much the same genetic structure as those within Whitsand Bay, which were estimated to yield almost exactly the allele frequency observed among D‐stage larvae. Finally, the nearest known populations of *M. edulis* are in the Tamar Estuary and the Fal Estuary, which are located 10 and 50 km to the east and west, respectively, of Whitsand Bay (Hilbish et al., 2003). They are too distant to be significant sources of early‐stage larvae (Gilg & Hilbish, 2003a, 2003b). Thus, we discount the possibility that the observed heterozygote deficiency is the result of population mixture.

Previous laboratory studies of larval survival suggest that non‐random mating is a distinct possibility for the observed heterozygotes deficiency. Bierne et al. (2002, 2006) found evidence of positive assortative mating among gametes, especially when sperm competition was allowed. Beaumont et al. (2004) did not find evidence of assortative mating among gametes, but their experimental design did not allow for sperm competition. Overall, these studies suggest that in nature, where sperm competition is expected to be present, there may be significant positive assortative mating that could yield an appreciable heterozygote deficiency. Strong positive assortative mating would explain how “pure” subpopulations of *Mytilus edulis* and *M. galloprovincialis* persist within the hybrid zone within southwest England (Diz & Skibinski, 2023).

Hilbish et al. (2012) found that the distribution of *Mytilus edulis* in France is correlated with regions where average winter sea surface temperature (SST) were <10–11°C. *M. galloprovincialis* prevailed in regions where winter average SST was >11°C and hybrid zones were centered on areas where there were an intermediate number of days in the winter with an SST near 10–11°C. One of the three hybrid zones they studied shifted it's position by over 100 km in an ~10 year period, which was correlated with warming in this region. Two other hybrid zones, located in regions that did not experience a change in the geography of these threshold temperatures, remained stable. The hybrid zone in southwest England has remained stable for over 40 years and recent winter SST in this region is near but has not exceeded the threshold temperature of 10–11°C (https://seatemperature.info). If warming SST in the winter leads to decreased performance by *M. edulis* and *M. edulis*‐like hybrids it would be expected that the hybrid zone in southwest England may be replaced by a population that is predominantly *M. galloprovincialis* and *M. galloprovincialis*‐like hybrids, similar to that currently observed in northern Cornwall (Figure 1).

In conclusion, this study provides the first evidence of directional selection during the larval stage that opposes the directional selection observed in adult hybrid populations of *Mytilus edulis* and *M. galloprovincialis*. This study illustrates the possibility that in organisms with complex life histories there may be different forms of selection operating in hybrid populations during different portions of the life cycle. Studies that seek to determine the forms of selection operating in hybrid zones should take this possibility into account. Additionally, it should be noted that studies that examine the net results of selection are potentially confusing multiple forms of selection occurring in different portions of the life cycle.

## AUTHOR CONTRIBUTIONS

**Allison B. Willis:** Conceptualization (equal); formal analysis (equal); investigation (equal); writing – original draft (equal); writing – review and editing (equal). **Evgeniya Ermolaeva:** Investigation (equal); writing – review and editing (equal). **Amaelia Zyck:** Investigation (equal); writing – review and editing (equal). **Rhiannon Rognstad:** Investigation (equal); methodology (equal). **Shannon Davis:** Methodology (equal). **Thomas J. Hilbish:** Conceptualization (equal); formal analysis (equal); funding acquisition (equal); investigation (equal); project administration (equal); supervision (equal); writing – original draft (equal); writing – review and editing (equal).

## CONFLICT OF INTEREST STATEMENT

The authors have no conflict of interest.

## Data Availability

The data underlying this article are available in the Dryad Digital Repository (DOI: 10.5061/dryad.xsj3tx9nt).
